# Efficacy of Intra-articular Platelet-Rich Fibrin Injection for Periarthritis of the Shoulder: A Pilot Study

**DOI:** 10.7759/cureus.103948

**Published:** 2026-02-20

**Authors:** Ankit Hooda, Hariprasad Seenappa, Tarun Kumar Somisetty Venkata Sai, Nagakumar J S., Nulaka Harish

**Affiliations:** 1 Department of Orthopedics, Sri Devaraj Urs Medical College, Sri Devaraj Urs Academy of Higher Education and Research, Kolar, IND; 2 Department of Orthopedics, R.L. Jalappa Hospital and Research Centre, Kolar, IND; 3 Department of Orthopedics and Traumatology, Sri Devaraj Urs Medical College, Sri Devaraj Urs Academy of Higher Education and Research, Kolar, IND

**Keywords:** adhesive capsulitis, autologous blood-derived orthobiologics, periarthritis shoulder, platelet-rich fibrin, regenerative medicine therapies, shoulder stiffness

## Abstract

Background

Periarthritis of the shoulder (adhesive capsulitis) is a common and disabling condition characterized by pain and progressive restriction of shoulder motion. Conventional treatment modalities often provide inconsistent long-term outcomes. Platelet-rich fibrin (PRF) is an autologous biological product with regenerative and anti-inflammatory properties, though evidence for its intra-articular use in periarthritis remains limited.

Objective

To evaluate the clinical outcomes and safety of a single intra-articular injectable PRF (i-PRF) injection in patients with periarthritis of the shoulder.

Methods

This prospective single-arm interventional pilot study included 30 patients with periarthritis of the shoulder of less than six months' duration. All patients received a landmark-guided intra-articular i-PRF injection (approximately 3-4 mL) prepared using low-speed centrifugation (700 RPM, 60g at radius 110 mm, 3 minutes). Outcomes were assessed using the visual analog scale (VAS), Shoulder Pain and Disability Index (SPADI), and shortened Quick Disabilities of the Arm, Shoulder, and Hand (QuickDASH) at baseline, immediate post-procedure, and at 2, 4, 8, and 12 weeks. Statistical analysis was performed using repeated measures analysis of variance (ANOVA).

Results

All patients completed the 12-week follow-up. Mean VAS scores improved significantly from 7.8 ± 1.2 at baseline to 1.5 ± 0.7 at 12 weeks (81% improvement, Cohen's *d* = 6.45, *P* < 0.001). SPADI scores improved from 68.5 ± 10.2 to 22.5 ± 6.3 (67% improvement, Cohen's *d* = 5.42), and QuickDASH scores from 65.3 ± 11.5 to 21.8 ± 7.1 (67% improvement, Cohen's *d* = 4.71) (*P* < 0.001 for both). Active range of motion improved significantly: forward flexion by 78.3°, abduction by 75.7°, and external rotation by 32.5° (all *P* < 0.001). Progressive improvement was observed at all follow-up intervals. No serious adverse events were reported.

Conclusions

Intra-articular PRF injection was associated with significant and sustained improvement in pain and functional outcomes in periarthritis of the shoulder, with an excellent safety profile. These preliminary findings support further randomized controlled studies to establish definitive efficacy.

## Introduction

The shoulder joint possesses the greatest range of motion among all human joints, enabling complex functional activities but also predisposing it to specific pathological conditions. Periarthritis of the shoulder (adhesive capsulitis) is characterized by chronic pain and progressive restriction of both active and passive glenohumeral movements [1]. The prevalence of periarthritis of the shoulder ranges from 3% to 5% in the general population and nearly 20% in individuals with diabetes mellitus [2]. It most commonly affects individuals between 40 and 70 years of age [3]. Pathophysiologically, the condition involves synovial inflammation followed by capsular fibrosis and contracture, leading to stiffness and functional limitation [4]. Conventional management strategies include physiotherapy, oral analgesics, intra-articular corticosteroid injections, hydrodilatation, and surgical interventions for refractory cases [5-7]. Although corticosteroid injections provide effective short-term pain relief, their benefits often diminish over time, and repeated administration may cause deleterious local and systemic effects, including cartilage degradation, tendon weakening, and hyperglycemia [8]. Platelet-rich fibrin (PRF) is a second-generation autologous platelet concentrate developed without anticoagulants. Injectable PRF (i-PRF) is a liquid formulation that allows gradual release of growth factors such as platelet-derived growth factor (PDGF), transforming growth factor-β (TGF-β), and vascular endothelial growth factor (VEGF), which may promote tissue healing and modulate inflammation [9,10]. Unlike corticosteroids, which provide symptomatic relief through immunosuppression, i-PRF promotes tissue regeneration and remodeling of the fibrotic capsule through growth factor-mediated mechanisms. Compared with platelet-rich plasma (PRP), i-PRF offers sustained cytokine release and a simplified preparation process without biochemical additives, while remaining in liquid form for easy injectability [11]. Despite increasing use of orthobiologics in musculoskeletal disorders, evidence regarding intra-articular i-PRF use in periarthritis of the shoulder remains sparse. This pilot study was therefore conducted to evaluate the clinical outcomes and safety profile of intra-articular i-PRF injection in patients with periarthritis of the shoulder. We hypothesized that a single i-PRF injection would result in clinically meaningful improvements in pain, functional scores, and shoulder range of motion at 12 weeks compared to baseline.

## Materials and methods

Study design and ethics

This was a prospective single-arm interventional pilot study conducted at the Department of Orthopedics, R.L. Jalappa Hospital, Kolar, from July 2024 to January 2025. Institutional Ethics Committee approval was obtained before study initiation (Approval no.: SDUAHER/ R&D/CEC/SDUMC-PG169/NF/2025-26). Written informed consent was obtained from all participants, and the study adhered to the Declaration of Helsinki.

Participants

Thirty consecutive patients fulfilling the inclusion criteria were enrolled. Sample size was determined based on feasibility considerations appropriate for a pilot study, with the primary aim of effect size estimation and procedure feasibility assessment.

Inclusion Criteria

The inclusion criteria were age 18-75 years, clinical diagnosis of periarthritis of the shoulder with symptom duration < 6 months, failure of at least three months of conservative treatment, and restriction of active and passive shoulder movements.

Exclusion Criteria

The exclusion criteria were prior shoulder trauma or surgery, hematological disorders or anticoagulant therapy, shoulder instability or neurological disorders, radiographic evidence of glenohumeral arthritis or full-thickness rotator cuff tear on ultrasound, local infection or history of septic arthritis, pregnancy or lactation, and prior corticosteroid injection within three months.

PRF Preparation

Twenty milliliters of autologous venous blood was collected from the antecubital vein under aseptic conditions in 10 mL sterile plain tubes (without anticoagulant) and centrifuged immediately at 700 RPM (approximately 60g relative centrifugal force at rotor radius of 110 mm) for three minutes using a calibrated centrifuge (Model- Remi R 303, Remi Elektrotechnik Limited, Mumbai, India). This low-speed centrifugation protocol produces i-PRF, which remains in liquid form. Following centrifugation, the supernatant liquid layer containing platelets and plasma was carefully aspirated using a sterile syringe, avoiding the red blood cell layer at the bottom. The resulting i-PRF (approximately 3-4 mL per 10 mL tube) was immediately loaded for intra-articular injection [12].

Injection Technique

Landmark-guided posterior glenohumeral injection was performed with the patient in a sitting position. The injection site was identified 2-3 cm inferior and 1-2 cm medial to the posterolateral corner of the acromion. After sterile skin preparation and local anesthetic infiltration, a 22-gauge spinal needle (9 cm) was inserted and directed anteriorly toward the coracoid process until glenohumeral joint entry was confirmed by lack of resistance. Approximately 3-4 mL of i-PRF was injected slowly. Landmark guidance was selected due to its routine use in outpatient orthopedic practice and documented acceptable accuracy of 70%-93% [13]. All patients were advised relative rest for 48 hours post-injection and instructed in a uniform set of pendulum and assisted range-of-motion exercises at the time of injection; however, formal monitoring of home exercise adherence was not performed.

Outcome measures

Assessments were performed by a single trained assessor at baseline, immediate post-procedure, and at 2, 4, 8, and 12 weeks using validated instruments.

Primary Outcomes

Visual analog scale (VAS): A 0-10 scale for pain intensity (0 = no pain, 10 = worst imaginable pain)

Shoulder Pain and Disability Index (SPADI) [14]: A 13-item questionnaire (0-100 scale), with higher scores indicating greater disability

Shortened QuickDASH [15]: An 11-item questionnaire assessing upper extremity function (0-100 scale)

Secondary Outcomes

Active range of motion measured using goniometry (forward flexion, abduction, external rotation at the side, and internal rotation measured by the vertebral level reached by the thumb behind the back) and adverse event monitoring at each visit

Statistical analysis

Data were analyzed using SPSS version 22.0 (IBM Corp., Armonk, NY). Normality was assessed using the Shapiro-Wilk test. Repeated measures ANOVA with Greenhouse-Geisser correction was used to compare outcomes across time points. Post hoc pairwise comparisons were performed using Bonferroni correction. Internal rotation data, being ordinal in nature (expressed as vertebral level reached), were analyzed separately using the Wilcoxon signed-rank test. Effect sizes were calculated using Cohen's *d* (baseline to 12 weeks). A *P*-value <0.05 was considered statistically significant. No missing data were encountered, as all patients completed follow-up.

## Results

Baseline characteristics

All 30 patients completed the study without loss to follow-up. Baseline demographic and clinical characteristics are presented in Table 1.

**Table 1:** Baseline patient characteristics (n = 30). SD, standard deviation; BMI, body mass index

Characteristics	Value
Age, years (mean ± SD)	52.4 ± 8.7
Female, *n* (%)	18 (60%)
Male, *n* (%)	12 (40%)
Affected side (right), *n* (%)	19 (63.3%)
Symptom duration, months (mean ± SD)	4.2 ± 1.3
Body mass index, kg/m² (mean ± SD)	26.3 ± 3.8
Dominant side affected, *n* (%)	21 (70%)
Comorbidities	
Diabetes mellitus	8 (26.7%)
Hypertension	11 (36.7%)
Hypothyroidism	4 (13.3%)
Hyperlipidemia	6 (20%)
Obesity (BMI ≥ 30 kg/m²)	5 (16.7%)
No comorbidities	9 (30%)

Primary outcomes

Significant progressive improvement was observed in all primary outcome measures across follow-up intervals (Table 2).

**Table 2:** Primary outcome measures over time (n = 30). *Repeated measures ANOVA. **Cohen's *d* comparing baseline to 12 weeks (values >0.8 indicate a large effect size). ANOVA, analysis of variance

Measure	Baseline	2 weeks	4 weeks	8 weeks	12 weeks	*P*-value*	Cohen's d**
VAS (0-10)	7.8 ± 1.2	6.2 ± 1.1	4.5 ± 1.0	2.8 ± 0.9	1.5 ± 0.7	<0.001	6.45
SPADI (0-100)	68.5 ± 10.2	58.3 ± 9.5	45.2 ± 8.7	32.8 ± 7.5	22.5 ± 6.3	<0.001	5.42
QuickDASH (0-100)	65.3 ± 11.5	56.7 ± 10.2	43.8 ± 9.3	31.5 ± 8.4	21.8 ± 7.1	<0.001	4.71

Secondary outcomes

Active range of motion showed significant improvement in all planes from baseline to 12 weeks (Table 3).

**Table 3:** Active range-of-motion outcomes (n = 30). *Internal rotation expressed as the median vertebral level reached.

Movement	Baseline (degrees)	12 weeks (degrees)	Mean improvement	*P*-value
Forward flexion	98.3 ± 15.6	176.7 ± 8.2	78.3 ± 14.8	<0.001
Abduction	87.5 ± 18.3	163.2 ± 12.5	75.7 ± 16.4	<0.001
External rotation	22.7 ± 8.5	55.2 ± 10.3	32.5 ± 9.2	<0.001
Internal rotation*	T12	T8	4 vertebral levels	<0.001

All improvements exceeded minimal clinically important differences reported in the literature (VAS: 1.4 points; SPADI: 13 points; QuickDASH: 14 points).

Subgroup analysis

Among eight diabetic patients, improvement trajectories were similar to those of non-diabetic patients. At two weeks, diabetic patients showed slightly less improvement in VAS scores (mean reduction, 1.2 points vs. 1.8 points in non-diabetic patients); however, by 12 weeks, outcomes were comparable (VAS reduction, 6.1 vs. 6.4 points; *P* = 0.52). This observation requires confirmation in larger studies.

Safety outcomes

No serious adverse events were reported during the study period. Three patients (10%) experienced mild transient post-injection discomfort (VAS 3-4) resolving spontaneously within 48 hours without intervention. No infections, neurovascular complications, or systemic reactions were observed.

## Discussion

This pilot study demonstrates that intra-articular i-PRF injection is associated with significant, progressive, and clinically meaningful improvements in pain, functional scores, and shoulder mobility inpatients with periarthritis of the shoulder over 12 weeks. The sustained improvement trajectory, with continued gains observed between 8 and 12 weeks, may be attributed to the gradual release of growth factors from the liquid i-PRF formulation, promoting sustained anti-inflammatory and regenerative effects [16,17]. It is important to emphasize that, given the absence of a control group, causal attribution of the observed improvements to i-PRF injection cannot be established; the findings should, therefore, be interpreted as hypothesis-generating rather than confirmatory.

Comparison with existing literature

Our findings align with previous studies evaluating platelet-based therapies in adhesive capsulitis. The VAS reduction from 7.8 to 1.5 (81% improvement) compares favorably with reported PRP outcomes. Kothari et al. reported VAS improvement from 6.8 to 2.1 (69% improvement) at 12 weeks with PRP versus 6.7 to 3.9 (42% improvement) with corticosteroids [18]. Similarly, Gupta et al. demonstrated superior mid-term outcomes with PRP compared to corticosteroids, with sustained benefits beyond three months [19]. Blanchard et al. reported 65% pain reduction with PRP at six months in adhesive capsulitis [20].

Mechanistic considerations

i-PRF offers theoretical advantages over both corticosteroids and PRP. Unlike corticosteroids, which provide symptomatic relief through anti-inflammatory mechanisms but may impair tissue healing, i-PRF delivers supraphysiological concentrations of growth factors in a liquid formulation that promotes angiogenesis, collagen synthesis, and tissue remodeling [9,10]. The PDGF and TGF-β released from i-PRF may facilitate organized capsular remodeling rather than simple inflammation suppression. The liquid nature of i-PRF allows for easier intra-articular distribution compared to solid PRF clots, potentially enhancing bioavailability throughout the joint space. Compared to PRP, i-PRF maintains sustained cytokine release while avoiding anticoagulants, reducing immunogenic risk, and simplifying clinical application [11,21].

Clinical significance and cost considerations

The magnitude of improvement observed (81% pain reduction, 67% functional improvement) substantially exceeds minimal clinically important differences and likely represents meaningful quality-of-life enhancement for patients. From a practical perspective, i-PRF preparation requires approximately 10-15 minutes and basic low-speed centrifugation equipment available in most orthopedic settings. The liquid formulation allows for easy aspiration and injection without clot manipulation. While initial setup costs exist, the autologous nature eliminates ongoing material costs beyond standard venipuncture supplies. Patient acceptance was excellent in our cohort, with the biologic nature of the intervention perceived favorably. Comparative cost-effectiveness studies against corticosteroids and physiotherapy would be valuable.

Study limitations

Several important limitations must be acknowledged. The absence of a control group limits causal inference; observed improvements may reflect placebo effects or concurrent physiotherapy rather than PRF efficacy specifically. However, it is worth noting that our inclusion criteria restricted enrollment to patients with symptom duration of less than six months, placing the cohort predominantly in the freezing phase of adhesive capsulitis, during which spontaneous improvement within a 12-week window is unlikely based on the well-established natural history of the disease. Nevertheless, the potential influence of natural disease progression and regression to the mean cannot be entirely excluded in the absence of a control group. The small sample size (*n* = 30) and single-center design limit generalizability. Landmark-guided injection technique, while clinically practical, has reported accuracy of only 70%-93% [13]; some injections may have been extra-articular, potentially underestimating true efficacy. The outcome assessor was not formally blinded throughout the study period, which may have introduced assessment bias. The 12-week follow-up duration, while adequate for pilot evaluation, does not establish long-term durability. While all patients received uniform pendulum and assisted range-of-motion exercise instructions at the time of injection, adherence to the home exercise program was not formally monitored, introducing potential variability in concurrent treatment. Selection bias from consecutive sampling may have preferentially included motivated patients. Future randomized controlled trials should address these limitations through control groups (saline placebo, corticosteroid, or PRP comparators), ultrasound-guided injections, blinded assessors, larger sample sizes, standardized concurrent therapy protocols, and follow-up extending to 6-12 months. As a pilot study, these findings primarily inform feasibility and effect size estimation for future definitive trials rather than establishing definitive efficacy. The excellent safety profile, high completion rate, and very large effect sizes (Cohen's *d* > 4.5) support the scientific and ethical justification for larger randomized controlled studies.

## Conclusions

Intra-articular PRF injection is associated with significant and sustained improvements in pain, function, and range of motion in patients with periarthritis of the shoulder over 12 weeks, with an excellent safety profile and effect sizes exceeding conventional minimal clinically important differences. These preliminary findings from a pilot study support the feasibility and potential efficacy of i-PRF as a treatment modality for adhesive capsulitis. Larger randomized controlled trials with sham or active comparators, ultrasound-guided injection techniques, longer follow-up periods, and standardized concurrent therapy protocols are required to confirm these findings and define i-PRF's role in routine clinical practice.
